# Supplementary material for: Structural basis for regulated assembly of the mitochondrial fission GTPase Drp1
Source: Nat Commun. 2024 Feb 13;15:1328. doi: 10.1038/s41467-024-45524-4 (PMC10864337; doi:10.1038/s41467-024-45524-4)
Supplement: Supplementary file 3 — Description of Additional Supplementary Files [file 41467_2024_45524_MOESM3_ESM.pdf]

Description of Additional Supplementary Materials

Movie 1: Cryo-EM dimer interface of Drp1 compared to the crystal structure. The starting position represents the crystal structure (PDB code 4BEJ). Morphing from this structure to the cryo-EM structure of Drp1 reveals a flexible, compressed interface compared to the crystal structure. Blue: stalk/middle domain; Red/Pink: BSE; Green: G Domain.

Movie 2: Compaction of the G Domains represent an auto-inhibited state. The starting position represents an open, active conformation observed in the crystal structure (PBD code: 4BEJ). Morphing from this structure to the native cryo-EM structure of Drp1 reveals a compaction of the G Domains (green) against the distal end of the stalk, representing a BSE lock, stabilizing an inactive conformation that is auto-inhibited.

Movie 3: A BSE lock regulates Drp1. In the inactive conformation, the BSE (Red) creates a lock at the distal end of the stock at loop 3. R456 (magenta) stabilizes this lock. By introducing a charge mutation, the protein is primed for assembly.

Movie 4: Y493 stabilizes the dimer interface. Y493 (light green) bookends the dimer interface acting as a shock absorber to accommodate an array of heterogeneity. Mutating this to Y493A destabilizes the dimer and breaks Drp1 ability to assemble into larger polymers.
